# The Quantity and Quality of Anxiety Are Mediating Variables between Motivation, Burnout and Fear of Failing in Sport

**DOI:** 10.3390/ijerph182312343

**Published:** 2021-11-24

**Authors:** Elisa I. Sánchez-Romero, Francisco J. Ponseti Verdaguer, Jaume Cantallops Ramón, Alejandro García-Mas

**Affiliations:** 1 Department of Social, Legal and Business Sciences, Catholic University of Murcia (UCAM), 30107 Murcia, Spain; eisanchez@ucam.edu; 2 Research Group of Sports Sciences (GICAFE), Department of Pedagogy, University of the Balearic Islands, 07122 Palma, Spain; xponseti@uib.es (F.J.P.V.); alex.garcia@uib.es (A.G.-M.)

**Keywords:** behavioural regulation, anxiety, burnout, self-determined motivation, fear of failure, athletes

## Abstract

The purpose of this study was to examine the effect mediated by self-determined motivation on an athlete’s burnout, anxiety and fear of failure. To this end, the sample group consisting of 235 semi-professional yachtsmen and women was asked to answer four questionnaires which set out to assess each of the study’s variables: Behavioural Regulation in Sport Questionnaire, Performance Failure Appraisal Inventory, Sport Anxiety Scale-2 and Athlete Burnout Questionnaire. The ex-post facto research was designed to analyse a single group based on structural equation modelling (SEM) between the constructs under analysis. The results illustrate that behavioural regulation could be used to positively predict burnout and fear of failure mediated by sport anxiety. Moreover, behavioural regulation has a significant, positive and moderate bearing on burnout and is wholly mediated by sport anxiety. It concludes by underscoring the point that there is no relationship either between behavioural regulation and burnout or between behavioural regulation and fear of failure, unless the athlete in question experiences anxiety.

## 1. Introduction

According to the taxonomy of the self-determination theory (SDT) [1], motivation is regarded as a continuum composed of six behavioural regulating factors, ranging from higher to lower self-determination: intrinsic motivation, extrinsic motivation (integrated regulation, identified regulation, introjected regulation and external regulation) and amotivation [2,3,4,5].

Deci and Ryan [1] defined intrinsic motivation as the pleasure one finds in the performance of an activity, which is why the activity in question is deemed to be an end in itself. On the other hand, extrinsic motivation denotes the motivation to take part in an activity with a view to achieve other goals, which is why it is mediated by rewards or external agents. In turn, it integrates various degrees of regulation: integrated regulation, identified regulation, introjected regulation and external regulation. Finally, amotivation is the lowest level of self-determination and denotes a total lack of motivation to take part in an activity, whether it be intrinsic or extrinsic. It manifests itself when an individual has no intention to act and does not value an activity.

The most self-determined form of extrinsic motivation is integrated regulation as it involves not only identifying with the importance of behaviour but also integrating these identifications with other aspects of the self. Regulations will not be integrated until the individuals have fully accepted them by striking a harmonious or coherent balance between them and other aspects of their values and identity [6].

Identified regulation is the process by which individuals acknowledge and accept the underlying value of a behaviour. By identifying with the value of a behaviour, individuals internalise the regulation more fully and accept it more fully as their own. The resulting behaviour is therefore more autonomous and tends to result in greater commitment and enhanced performance.

Introjected regulation is linked to expectations about ego, self-approval, prevention of anxiety and improving ego strength in aspects such as pride. Behavioural regulation continues to have a locus of external control [7]. The individual who has an external locus of control is more dependent on other people’s opinions when making decisions, and they are less resistant to the difficulties and display with no constructive attitude in performance [8].

External regulation represents the least self-determined level of extrinsic motivation and therefore contrasts most starkly with intrinsic motivation (it emerges from the individual himself). This form of regulation is characterised by a locus of external control whose purpose is either to satisfy an external demand or to obtain a reward; it is negatively related not to performance or persistence, but to a decline in well-being [9].

Each of the aforementioned degrees of behavioural self-regulation has its own structure and is regulated by the subject either internally or externally; the highest degrees of self-regulation are reached on account of the subject’s intrinsic motivation [10]. The processes regulating motivation may take the form of values, rewards, self-control, interests, enjoyment, satisfaction, etc. In a sporting context, the SDT framework is useful as it explains the motivational, cognitive and emotional processes of athletes [11]. 

One of the most widely analysed variables in sport is anxiety, which has traditionally been assigned to one of two categories: state anxiety or trait anxiety. In sport, state anxiety may occur immediately before or during a competition [12]. Anxiety comprises three factors: one is the emotional and somatic state which is characterised by apprehension and tension associated with the activation of the body [13]. Competitive anxiety also comprises two cognitive factors. On the one hand, worry denotes a concern about the potentially negative consequences associated with a poor performance; on the other hand, concentration disruption occurs when an athlete finds it difficult to concentrate on key aspects of the task at hand, with the ensuing inability of the athlete to think clearly in a competitive situation [14]. Trait anxiety refers to prolonged periods of anxiety in which the individual tends to react anxiously to stimuli that, in the absence of anxiety, do not usually cause stress [15]. 

Burnout is another negative variable and is one of the most debilitating states that an athlete can experience. It occurs following a continuous exposure to the chronic effects of stress [16]. It is characterised by emotional and/or physical exhaustion and a reduced sense of accomplishment and sport devaluation in general, caused by the exacting demands of training and competition [16]. Burnout is negatively related to self-esteem [17] and resistance [18]. Conversely, it is positively related to physical and emotional discomfort [19], doping [20], and reduced sports performance [21].

On the other hand, athletes generally exhibit a fear of failure and evaluate their own performance [22]. The fear of failure stems from an athlete’s inability to appropriately manage and control certain sporting situations (e.g., fear of failure appears to energise behaviour and biases individuals toward the pursuit of avoidance achievement goals and reduces sub-optimal or dysfunctional achievement motivation) [23], thereby causing the athlete to experience a high degree of anxiety with various consequences (a feeling of shame, lower self-esteem, fear of an uncertain future, others losing interest and/or upsetting others) [24], as a result of which they may adopt problems with social relations and behaviour and lack of interest [25]. In relation with that, recent findings show a robust negative correlation between social competence and behavioural problems, outlining the need to consider both variables as related and globally when an intervention in that sense was planned [26].

The most intense and widely analysed psychological variables in sport are characterised by two aspects: “positive” (which promotes psychological well-being, healthy sporting practice and, in some cases, effective performance) and “negative” (linked to a loss of concentration or motivation). From this negative perspective, for the studies focusing predominantly on practical application, training or psychological intervention, the most noteworthy variables selected for this research are related to self-determined motivation, competitive anxiety, fear of failure or making mistakes and burnout. In a recent study about well-being in sport, the most noteworthy aspect is the influence of adversity factors on the performance of professional athletes [27]. In respect of preventing negative results both for the athlete and the individual in general, all these factors are increasing in relevance. 

Applied sport psychology is now shifting from an approach of intervention to one of prevention. To this end, it is necessary to conduct empirical and/or experimental studies with a primary view of predicting both positive and negative psychological effects.

That is why this study sets out to analyse the relationship between the least self-determined levels of behavioural regulation in sport (hereinafter referred to as behavioural regulation) and burnout, and between behavioural regulation and the fear of failure during a sporting performance (hereinafter referred to as fear of failure), in a bid to describe the mediating role of sport anxiety. The following hypotheses are considered: (1) Behavioural regulation has a direct (positive) bearing on burnout; (2) Behavioural regulation has a direct (positive) effect on the fear of failure; (3) Behavioural regulation has a direct (positive) bearing on sport anxiety; (4) Behavioural regulation has an indirect bearing on burnout mediated by sport anxiety; and (5) Behavioural regulation has an indirect bearing on the fear of failure mediated by sport anxiety.

## 2. Materials and Methods

### 2.1. Design

The ex-post facto research was designed to analyse a single group [28] with a view to verifying the mediation of sport anxiety in relationships between behavioural regulation and burnout and between behavioural regulation and the fear of failure in semi-professional yachtsmen and women. This design enables a predictive model to be applied as its objective focuses on the study of existing relationships between variables [29].

### 2.2. Participants

The sample, based on intentional non-probability sampling, consisted of 235 semi-professional yachtsmen (146) and women (89) from the 49er category, who were aged between 16 and 52 (*M* = 24.66; *SD* = 8.03) and belonged to various sailing clubs based in the Autonomous Community of Andalusia (Spain). After the approval of this research’s plan from the Bioethics Committee for Human Research at Almería University (Ref. UALBIO 2019/014), the researcher team contacted various sailing clubs across Andalusia, asking for permission to distribute the questionnaires to their athletes. After explaining the main aims of this study to the participants, all of them were asked to fill in and sign an informed consent form (as for minors, their parents or legal guardians were asked for consent). The questionnaires were distributed under the supervision of a survey expert belonging to the research group, and a standardised procedure was used for guaranteeing that the instructions were the same for all the participants. Participants were given approximately 25 min to complete the questionnaire. To be selected to take part in the study, participants were required to volunteer, fill in the informed consent form and complete all the questionnaires.

### 2.3. Instruments

The following instruments have been used to carry out this study:

Introjected Regulation, External Regulation and Amotivation. Behavioural Regulation in Sport Questionnaire (BRSQ) [30]. As with the other measuring instruments, the Spanish version was used [31]. This scale consists of 36 items divided into six dimensions including intrinsic motivation, integrated regulation, identified regulation, introjected regulation, external regulation and amotivation. The items belonging to the final three dimensions were used for the purposes of this study: Introyected Regulation, External Regulation and Amotivation. All items share the root “I practise this sport…” followed by the differential content per sub-scale. Introyected Regulation (e.g., “because I would feel ashamed if I quit”), External Regulation (e.g., “because if I don’t other people will not be pleased with me”) and Amotivation (e.g., “but I question why I continue”). Items are valued from 1 (completely false) to 7 (completely true). This study produced Cronbach’s alpha values of *α* = 0.82 for introjected regulation, *α* = 0.85 for external regulation and *α* = 0.77 for amotivation. In respect of Viladrich et al. [29], values of *α* = 0.73, *α* = 0.62 and *α* = 0.71 were produced.

Sport Anxiety. Sport Anxiety Scale-2 (SAS-2) [32], which evaluates the anxiety that athletes experience when they are involved in a competitive situation. We used the Spanish version [31]; it consists of 15 items divided into three dimensions: Somatic Anxiety, Worry and Concentration Disruption, with five items allocated to each dimension. Somatic Anxiety (e.g., “My body feels tense”), Worry (e.g., “I worry that I will not play well”) and Concentration Disruption (e.g., “It is hard to concentrate on the game”). Every item began with “Before or while I play or compete…”. The answers were expressed according to a 4-point Likert scale ranging between 1 (nothing) and 4 (a lot). Ramis et al. [33] produced Cronbach’s alpha values of 0.83 for the somatic scale, 0.78 for the worry scale and 0.73 for the concentration disruption scale. Similar results were obtained for this study: *α* = 0.85 for the somatic scale, *α* = 0.88 for the worry scale and *α* = 0.81 for the concentration disruption scale.

Burnout. Athlete Burnout Questionnaire (ABQ) [34]. The Spanish version of ABQ was administered [35]. The instrument consists of 18 items divided into three dimensions (Physical Exhaustion, Reduced Sense of Accomplishment and Sport Devaluation), with six items allocated to each dimension: Physical Exhaustion (e.g., “I am exhausted by the mental and physical demands of [sport]), Reduced Sense of Accomplishment (e.g., “I’m accomplishing many worthwhile things in [sport]) and Sport Devaluation (e.g., “I have negative feelings toward [sport]). The answers to the instrument were expressed according to a Likert scale, from 1 (almost never) to 5 (almost always). The instrument presents Cronbach’s alpha values of 0.87, 0.71 and 0.82 for the three dimensions, respectively, with the following values produced for this research: *α* = 0.88 for physical exhaustion, *α* = 0.72 for reduced sense of accomplishment and *α* = 0.81 for sport devaluation.

Fear of Failure during a Sporting Performance. Performance Failure Appraisal Inventory (PFAI) [25]. The Spanish version [24] was applied. It consists of 25 items divided into five dimensions: Fear of Experiencing Shame (example item: “When I am not succeeding, I am less valuable than when I succeed”), Fear of Devaluing One’s Self-estimate (e.g., “When I am failing, I blame my lack of talent”), Fear of Having an Uncertain Future (e.g., “When I am failing, my future seems uncertain”), Fear of Important Others Losing Interest (e.g., “When I am not succeeding, people tend to leave me alone”) and Fear of Upsetting Important Others (e.g., “When I am failing, important others are not happy”). Every item began with “When I practise my sport…”. The answers were expressed according to a 5-point Likert scale ranging between 1 (I do not believe it at all) and 5 (I believe it 100%). This study produced Cronbach’s alpha values of *α* = 0.86 for fear of experiencing shame, *α* = 0.73 for fear of devaluing one’s self-estimate, *α* = 0.79 for fear of having an uncertain future, *α* = 0.85 for fear of important others losing interest and *α* = 0.82 for fear of upsetting important others; these values are very similar to those referenced by Moreno-Murcia and Conte [24], with *α* = 0.82, *α* = 0.69, *α* = 0.80, *α* = 0.85 and *α* = 0.93, respectively.

### 2.4. Procedure

For the purposes of the study, the Bioethics Committee for Human Research at Almería University (Ref. UALBIO 2019/014) was asked to approve the plan, which involved contacting various sailing clubs across Andalusia and asking for permission to distribute the questionnaires to their athletes. They were previously informed of the objectives of the study to ensure they had access to all necessary information. Before the scales were administered, all participants were asked to fill in and sign an informed consent form. As for minors, their parents or legal guardians were asked for consent. The questionnaire was distributed under the supervision of a survey expert belonging to the research group, who provided explanations and answered questions during its completion. Participants were given approximately 25 min to complete the questionnaire.

### 2.5. Statistical Analysis

The descriptive statistics (median, standard deviation) and Pearson’s correlation coefficients between the study’s variables were initially calculated. Cronbach’s alpha was also applied to measure the internal consistency of the dimensions of the scales used. For the purpose of verifying the individual reliability of each indicator with its respective construct used in the explanatory model proposed in this research, Exploratory Factor Analysis (EFA) was conducted on the basis of the maximum likelihood and varimax rotation extraction method [36]. All these analyses were conducted using IBM SPSS Statistics program (IBM, SPSS v.25.0, Armonk, NY, USA).

For verifying the reliability of scales, the Alpha statistic of the four constructs was calculated. Subsequently, to estimate the latent variables based on observable variables, Confirmatory Factor Analysis (CFA) was carried out, based on the maximum likelihood parameter estimation method, using the following indices to assess the model fit: chi-square/degrees of freedom (*χ^2^/df*), goodness of fit index (GFI), AGFI (adjusted goodness of fit index), normed fit index (NFI), Tucker–Lewis index (TLI), comparative fit index (CFI) and root mean square error of approximation (RMSEA). For validity analysis purposes, the composite reliability (CR) and average variance extracted (AVE) were calculated for every factor in the case of convergent validity, while maximum shared variance (MSV) and the square root of AVE were calculated for divergent validity analysis.

Structural equation analysis was also carried out from the perspective of structural equation models (SEM). The model used behavioural regulation, measured via the BRSQ, as an exogenous variable (one factor, with three indicators), sport anxiety, measured via the SAS-2, as a mediating variable (one factor, three indicators), and two endogenous variables: burnout, measured via the ABQ (one factor, three indicators), and fear of failure, measured via the PFAI (one factor, five indicators). Both the AFC and SEM were produced using IBM SPSS Amos Graphics, version 22 [37].

## 3. Results

### 3.1. Descriptive Statistics and Correlations

Table 1 shows the means and standard deviations of every dimension of the study. Of all the components involved in behavioural regulation in sport, measured on a scale of 1–7, introjected regulation yields the highest mean (*M* = 3.50; *SD* = 1.871), while external regulation yields the lowest (*M* = 2.67; *SD* = 1.727). In respect of sport anxiety, measured on a scale of 1–4, the worry scale yields the highest mean (*M* = 2.85; *SD* = 0.862) while the concentration disruption scale yields the lowest (*M* = 2.09; *SD* = 0.730). As for burnout dimensions, measured on a scale of 1–5, the reduced sense of accomplishment dimension yields the highest mean (*M* = 2.87; *SD* = 0.610) while sport devaluation yields the lowest (*M* = 1.79; *SD* = 0.902). Finally, regarding the fear of failure, the factor concerning the fear of experiencing shame yields the highest mean (*M* = 2.34; *SD* = 0.993), whereas the factor concerning the fear of upsetting important others yields the lowest (*M* = 1.90; *SD* = 0.879).

The same table shows that almost all Pearson correlations between dimensions have produced statistically significant values, except for the relationship between behavioural regulation in sport (three dimensions) and worry mediated by anxiety, and between behavioural regulation and a reduced sense of accomplishment of burnout; between worry mediated by anxiety and the sport devaluation dimension of burnout; and between external regulation and the fear of experiencing shame, and between external regulation and fear of devaluing one’s self-estimate. All resulting values are positive, except for the relationships between the scale of worry mediated by anxiety and the two least self-determined levels of motivation. The highest value in absolute terms is yielded by the relationship between introjected regulation and external regulation (*r_xy_ =* 0.779) while the lowest value is yielded by the relationship between the dimension of worry mediated by sport anxiety and amotivation (*r_xy_ =* −0.053). 

Finally, the main diagonal of the matrix of Table 1 presents the values of Cronbach’s alpha coefficients for each of the dimensions under consideration; they are satisfactory for all dimensions as they are above the threshold of 0.70 (between 0.72 and 0.88). Moreover, the four constructs of this research yielded acceptable Cronbach’s alpha values: behavioural regulation *α* = 0.87, sport anxiety *α* = 0.71, burnout *α* = 0.70 and fear of failure *α* = 0.91. 

### 3.2. Exploratory Factor Analysis

In order to determine the unidimensionality of the constructs considered in the questionnaire for the explanatory model proposed in this research, every single construct was assessed by exploratory factor analysis (EFA). The EFA method involves principal components with Varimax rotation based on the criterion of eigenvalues being greater than 1. 

Table 2 shows the factor analysis of constructs. In respect of all constructs, the EFA is valid, as measurements produced according to the Kaiser–Meyer–Olkin (KMO) sampling adequacy method are greater than 0.65 in all cases, and *χ*^2^ values are significant (*p* < 0.000) with just one factor with an eigenvalue greater than 1, which explains more than 60% of the variance and with factor loadings greater than 0.5 (even 0.7). 

### 3.3. Confirmatory Factor Analysis

**Figure 1:**
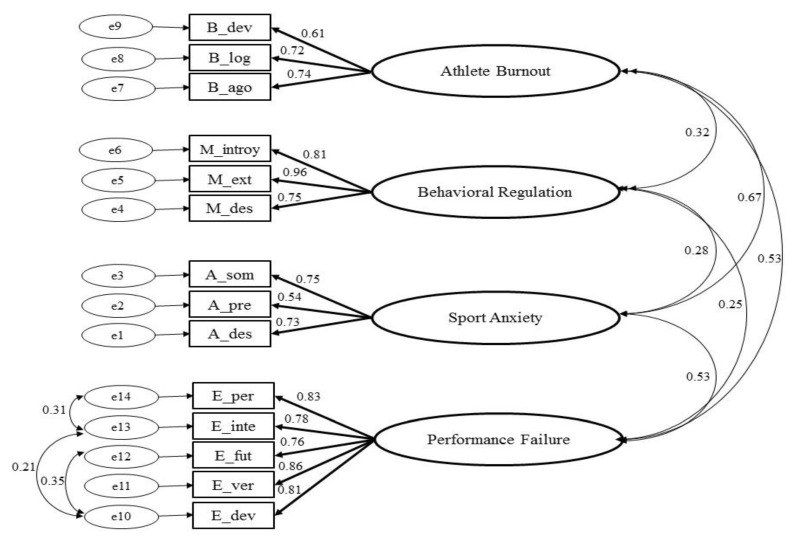
CFA with standardised coefficients and correlations between constructs.

The results of the model produced by CFA are featured in Figure 1. This model represents a factorial structure consisting of four constructs or latent variables (behavioural regulation, sport anxiety, burnout and fear of failure) and 14 observable variables (items or indicators of latent variables), which show the factor loadings between constructs and observable variables, as well as the correlations between constructs. From the resulting model, the observable variables for each construct present suitable factor loadings, with values significantly different from zero and greater than 0.5, with the lowest value being 0.61 (sport devaluation factor of burnout) and the highest being 0.96 (external motivation factor of behavioural regulation). This produced an average variance extracted of 71.4% in the case of behavioural regulation in sport, 46.2% in the case of sport anxiety, 48.0% in the case of burnout, and 65.8% in the case of fear of failure (Table 3).

Table 3 shows that composite reliability is greater than 0.7, and that AVE is greater than 0.5 for all constructs except burnout and sport anxiety. Although their AVE is less than 0.5, they are deemed to be valid due to their proximity to the acceptance threshold (0.480 and 0.462, respectively), which is why the model demonstrates convergent validity [38]. Moreover, all t values are significant at 95% (even at 99%), and the signs of all coefficients are positive, which is why every observable variable is directly related to the dimension with which it is associated.

The following condition must be fulfilled in order to demonstrate divergent validity: (1) MSV must be less than AVE and (2) the square root of AVE must be greater than the correlations between constructs [38,39]. As shown in Table 4, this condition is fulfilled by all constructs, which is why divergent validity is also assumed.

Table 5 shows the values recorded in the fit indices used on the basis of the maximum likelihood parameter estimation method. In the case of *χ^2^/df*, values below 5 (39) and even below 4 [40] are deemed to be acceptable, and the model proposed in this research presents acceptable values (χ^2^ (245.9)/df (68) = 3.616); in the case of the GFI, values below 0.85 are deemed to be acceptable, and the model presents a value of 0.854; as for AGFI values, they are acceptable above 0.80 (0.775); in the case of CFI, values above 0.90 (0.901) are deemed to be acceptable; and in the case of RMSEA, the values are below 0.1 (0.106) [40]. The proposed structural model presents an acceptable fit, as three of the five quality indices (GFI, CFI, *χ^2^/df*) are within the acceptance thresholds used for model validation purposes, while the other two (AGFI, RMSEA) present values near to the thresholds, which are deemed to be acceptable.

### 3.4. SEM

**Figure 2:**
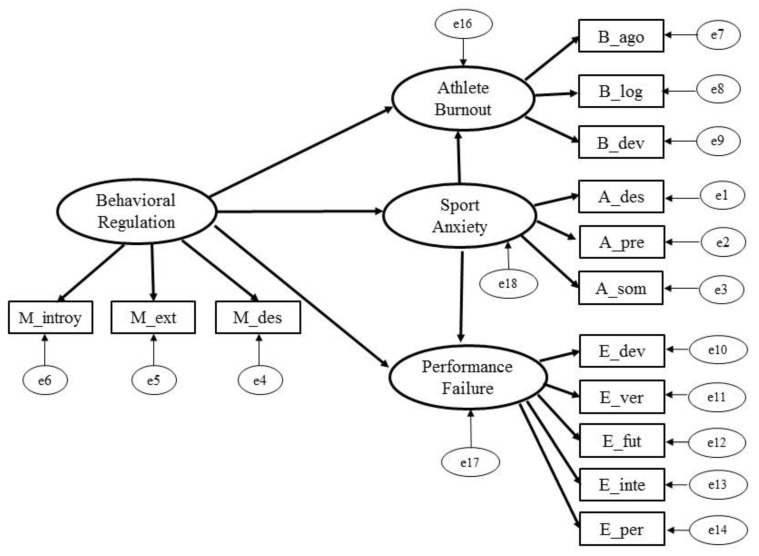
Hypothetical model among Behavioural Regulation, Sport Anxiety, Athlete Burnout and Performance Failure.

The initial hypothetical model considered that behavioural regulation would be able to positively predict burnout and fear of failure, mediated by sport anxiety (Figure 2). This general model was tested on a confirmatory basis and identified a number of satisfactory fit indices. 

**Figure 3:**
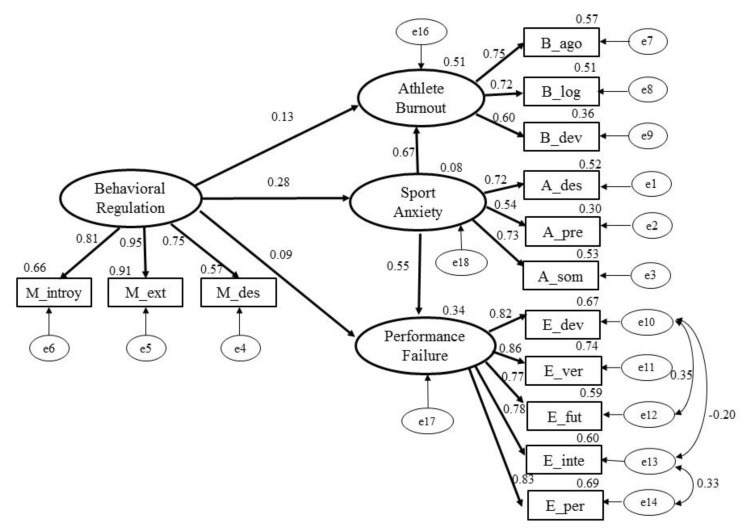
Structural model of Behavioural Regulation, Sport Anxiety, Athlete Burnout and Performance Failure.

The referenced model is featured in Figure 3. It contains 105 different sampling moments, 36 parameters to estimate and 69 degrees of freedom. The maximum likelihood method was used to estimate parameters, while significance contrasts in relation to direct, indirect and total effects were measured by bootstrapping. 

The indicators of the overall model fitness were as follows: *χ^2^*_(69)_ = 252.205 (*p* < 0.001), *χ^2^/df* = 3.655 (<5), GFI = 0.854 (>0.85), AGFI = 0.778 (<0.80), CFI = 0.906 (>0.90), and RMSEA = 0.107 (<0.1) [41]. The structural model therefore presents an acceptable fit as the quality indices are within, or very close to, the acceptance thresholds used for validation purposes [36]. Regression coefficients were statistically significant (*p* < 0.01), except for behavioural regulation with burnout (0.13) and with fear of failure (0.09). 

On the other hand, Table 6 presents values of the total effects between constructs, direct and indirect. In respect of contrasted relationships: (1) In accordance with hypothesis 1, behavioural regulation has no significant bearing on burnout (coefficient 0.13, *p* > 0.1); (2) In accordance with hypothesis 2, behavioural regulation has no significant bearing on fear of failure (coefficient 0.09, *p* > 0.1); (3) In accordance with hypothesis 3, behavioural regulation has a significant bearing on sport anxiety (0.278, *p* < 0.01); (4) In accordance with hypothesis 4, behavioural regulation has a significant, positive and moderate bearing on burnout, completely mediated by sport anxiety. It presents an indirect effect of 0.185 (*p* < 0.01) and a total effect (i.e., direct + indirect) of 0.319 (*p* < 0.01), with a proportion of variance in relation to burnout explained by the model of 0.51. In accordance with hypothesis 5, behavioural regulation has a significant, positive and moderate-high bearing on fear of failure, which is completely mediated by sport anxiety. It presents an indirect effect of 0.153 (*p* < 0.01) and a total effect (i.e., direct + indirect) of 0.246 (*p* < 0.01), with a proportion of variance in relation to fear of failure explained by the model of 0.34. 

In conclusion, in respect of hypotheses 4 and 5, the mediation of sport anxiety on relationships between behavioural regulation and burnout and between behavioural regulation and fear of failure was analysed in detail. There is complete mediation of anxiety as regards the two relationships under consideration, i.e., there is no relationship between behavioural regulation and burnout or between behavioural regulation and fear of failure unless the athlete in question experiences anxiety (Table 7). 

## 4. Discussion

Firstly, the results produced by this study confirm that the athlete’s personal experience of taking part in competitive sport is much more relevant than the dependence on external factors, even if they are very close and significant, such as family members [41].

Secondly, the findings duly confirm that the self-determination of players is based on introjection (internalisation of motivation accompanied by any “negative” emotion such as guilt, caused, for instance, by a situation of externally regulated motivation, as per the significant correlational data obtained, as explained extensively trough the SDT theory, e.g., [42]), which has been also linked empirically with competitive anxiety when the athlete, or the person who has developed a physical activity career, reacts to a situation including distress [43,44]; competitive anxiety caused by a concern about one’s own performance; and the most relevant burnout factor (with lower middle values, which was to be expected given the sample group and its age and experience), which is a sense of failing to achieve goals.

After these main discoveries were made, the model was tested to demonstrate relationships between variables as demonstrated by these data, to one degree or another, in contrast to those produced by previous studies which show that psychosocial factors (families, coaches, team-mates) have a much greater bearing on motivation, in particular, and on other psychological variables related to player performance [20,25]; although other studies do not shed so much light on this factor, let alone as consequences or factors of a single continuum [45,46], they suggest the opposite, a highly consistent and predictable order [9].

As we further analysed the results, we found, firstly, that both the first exploratory phase and the subsequent confirmatory phase of the proposed model produce very solid values which, along with the previously assessed reliability study, support the hypotheses considered in the objectives of the study.

In addition to that, and in relation with the previously assessed descriptive results, it is possible to assert that competitive anxiety plays a very important role as a mediating variable between the form of motivation and those which adversely affect performance and the self-concept of players (fear of failure and burnout).

This result is undoubtedly very interesting, as previous studies based on the same theoretical three-dimensional model of anxiety suggest that the performance worry factor is the least influential, and it usually has the highest values [12,35], but it is consistent in that it impacts—by mediation—the most personal and autonomous factors of the most relevant “negative” effects, such as fear of failure and burnout (as shown above).

As a collateral result comment, it can therefore be stated that there is a connection between the autonomous form of motivation most affected by a negative emotion (as shown, this may be guilt) [47], competitive anxiety based on a personal worry about performance and more personal factors of fear of failure and burnout (perception of another negative variable: shame in this case, and the perception of failing to achieve the personal goals in relation to others). While this finding, confirmed by the hypotheses proposed for the model, is, in theory, consistent with the governing principles of the SDT, it raises serious framing problems in terms of the “negative” findings discussed above.

Finally, this line of reasoning, which is expressed primarily by the empirical observations of the “dark side” of the personality of elite athletes (under review), must be further underpinned before it can be added to the theoretical corpus on which this study is based.

### 4.1. Conclusions and Highlights

The emotion of shame, which we may associate with the perception of incompetence (one of the basic needs to be satisfied, according to the classic SDT) is the most powerful of all those studied regarding the loss of sports performance.

Anxiety associated with competition plays a fundamental role mediating cognitions and performance, in a non-intuitive way, which is of great interest in psychological intervention in sports.

Burnout cannot be considered as the end of a continuous process, given its relationship with abandonment and continuation of sports practice in distress situations.

The concept of socially considered “negative” emotions should be reconsidered, taking into account sports performance and the athlete’s own perception.

### 4.2. Limitations of the Study

While the practical derivatives of the results are very interesting from a training perspective (and its psychological consideration), they may also raise an ethical and, by extension, a deontological problem: to what extent must an athlete perceive and feel guilty in order to internalise motivation and therefore become self-determined? How much external control by psychosocial factors, fundamentally coaches, is deemed to be necessary to “prevent” negative aspects of burnout and the perception of a fear of failure? Moreover, any other directly practical aspects, such as the completely secondary consideration of the somatic anxiety factor for practical purposes, which is normally cited by external observers as proof of “debilitating” nervousness, have been repeatedly found to be irrelevant [48].

As the selected sport and level of competition are semi-professional, negative aspects may not be exhibited excessively. Additionally, the very broad age range must also be taken into consideration.

This study was not designed to examine or monitor an extensive period of time, which is why it cannot assess the probability of occurrence, for instance, of burnout. 

Furthermore, the intentional non-probability sampling of this study is a type of sampling that is easier and cheaper to access, but it has a higher risk of sampling bias, which occurs when some members of a population are systematically more likely to be selected in a sample than others. Sampling bias limits the generalisability of findings because it is a threat to external validity, specifically population validity.

Finally, we cannot generalise the results to other athletes because of the peculiarity of the sport of sailing, due to its high socioeconomic profile, and because the athletes participating in this study all belong to the same geographical location. For this reason, it is necessary to continue investigating the relationships between the variables of this study in other athletes. In addition, it was found that anxiety turned out to be a variable with total mediation in the relationships between the other variables, so that there is no relationship between behavioural regulation and burnout and between behavioural regulation and performance failure if not through sport anxiety.

### 4.3. Further Developments

In view of the results and their critical assessment, it is possible to conclude that one of the subsequent studies to be produced is the critical point of transition (despite the hierarchical model of [42]; it excludes the existence of a continuum between the factors of self-determined motivation) from external regulation of sporting behaviour, to intrinsic regulation by way of introjection: similar to the deontological consideration specified above, is the perception of guilt inherent to that internalisation, which is necessary, according to the prevailing paradigm, to produce an acceptable level of self-determination? 

Another interesting development related with this study’s results would be to analyse the same model in a selected ad hoc sample that is passing through more negative and distressing situations, in order to find the fitness of the model in a context more prone to negative emotions.

## Figures and Tables

**Table 1:** Descriptive statistics and Pearson correlations.

	*M* (*SD*)	1	2	3	4	5	6	7	8	9	10	11	12	13	14
1. M_introy	3.50(1.871)	0.82	0.779 **	0.599 **	0.138 *	0.091	0.283 **	0.132 *	0.124	0.369 **	0.204 **	0.175 **	0.258 **	0.233 **	0.208 **
2. M_ext	2.67(1.727)		0.85	0.721 **	0.185 **	−0.037	0.271 **	0.172 **	0.106	0.455 **	0.110	0.110	0.245 **	0.222 **	0.240 **
3.M_des	2.70(1.733)			0.77	0.186 **	−0.053	0.243 **	0.174 **	0.054	0.404 **	0.219 **	0.163 *	0.316 **	0.291 **	0.303 **
4. A_som	2.13(0.849)				0.85	0.443 **	0.541 **	0.364 **	0.337 **	0.322 **	0.295 **	0.338 **	0.307 **	0.245 **	0.298 **
5. A_pre	2.85(0.862)					0.88	0.367 **	0.183 **	0.297 **	0.058	0.418 **	0.432 **	0.293 **	0.206 **	0.232 **
6. A_des	2.09(0.730)						0.81	0.387 **	0.320 **	0.457 **	0.328 **	0.265 **	0.277 **	0.273 **	0.341 **
7. B_ago	2.29(0.990)							0.88	0.591 **	0.421 **	0.244 **	0.264 **	0.225 **	0.300 **	0.279 **
8. B_log	2.87(0.610)									0.374 **	0.350 **	0.364 **	0.287 **	0.305 **	0.332 **
9. B_dev	1.79(0.902)									0.81	0.253 **	0.247 **	0.311 **	0.473 **	0.428 **
10. E_ver	2.34(0.993)										0.86	0.714 **	0.760 **	0.540 **	0.655 **
11. E_dev	2.20(0.947)											0.73	0.632 **	0.682 **	0.703 **
12. E_fut	2.06(1.073)												0.79	0.573 **	0.682 **
13. E_inte	1.93(0.954)													0.85	0.766 **
14. E_per	1.90(0.879)														0.82

Note. * *p* < 0.05; ** *p* < 0.01; Cronbach´s alpha in diagonal matrix. M_introy = introjected regulation; M_ext = external regulation; M_des = amotivation. A_som = somatic anxiety; A_pre = worry; A_des = concentration disruption. B_ago = physical exhaustion; B_log = reduced sense of accomplishment; B_dev = sport devaluation. E_ver = fear of experiencing shame; E_dev = fear of devaluing one’s self-estimate; E_fut = fear of having an uncertain future; E_inte = fear of important others losing interest; E_per = fear of upsetting important others.

**Table 2:** Exploratory factor analysis of the constructs.

	Behavioural Regulation	Sport Anxiety	Burnout	Performance Failure
M_introy	0.935			
M_ext	0.887			
M_des	0.860			
A_som		0.843		
A_pre		0.739		
A_des		0.804		
B_ago			0.852	
B_log			0.830	
B_dev			0.719	
E_ver				0.870
E_dev				0.855
E_fut				0.850
E_inte				0.828
E_per				0.889
% explained variance	63.5%	80.1%	64.4%	73.7%
KMO test	0.652	0.694	0.645	0.834
Barlett test	137.8 ***	388.4 ***	152.2 ***	811.9 ***

Note. *** *p* < 0.001. M_introy = introjected regulation; M_ext = external regulation; M_des = amotivation. A_som = somatic anxiety; A_pre = worry; A_des = concentration disruption. B_ago = physical exhaustion; B_log = reduced sense of accomplishment; B_dev = sport devaluation. E_ver = fear of experiencing shame; E_dev = fear of devaluing one’s self-estimate; E_fut = fear of having an uncertain future; E_inte = fear of important others losing interest; E_per = fear of upsetting important others. KMO = Kaiser–Meyer–Olkin.

**Table 3:** Dimensionality and convergent validity of measurement scales.

		Standardised Coefficients	*t-Student*	*p*
Behavioural Regulation	M_introy	0.814	12.959	***
	M_ext	0.955	13.657	***
	M_des	0.753	Reference category	
	Composite Reliability	88.1%		
	Variance extracted	71.4%		
Sport anxiety	A_som	0.746	8.644	***
	A_pre	0.543	6.982	***
	A_des	0.732	Reference category	
	Composite Reliability	71.7%		
	Variance extracted	46.2%		
Athlete burnout	B_dev	0.609	7.816	***
	B_log	0.721	8.777	***
	B_agot	0.741	Reference category	
	Composite Reliability	73.3%		
	Variance extracted	48.0%		
Performance failure	E_per	0.832	13.750	***
	E_inte	0.785	11.678	***
	E_fut	0.764	15.798	***
	E-ver	0.858	14.318	***
	E_dev	0.814	Refrence category	
	Composite Reliability	90.6%		
	Variance extracted	65.8%		

Note. *** *p* < 0.01. M_introy = introjected regulation; M_ext = external regulation; M_des = amotivation. A_som = somatic anxiety; A_pre = worry; A_des = concentration disruption. B_ago = physical exhaustion; B_log = reduced sense of accomplishment; B_dev = sport devaluation. E_ver = fear of experiencing shame; E_dev = fear of devaluing one’s self-estimate; E_fut = fear of having an uncertain future; E_inte = fear of important others losing interest; E_per = fear of upsetting important others.

**Table 4:** Divergent validity of constructs.

				Correlations (Variances)			
	CR	AVE	MSV	1	2	3	4
1. Athlete burnout	0.733	0.480	0.446	0.693			
2. Sport anxiety	0.717	0.462	0.446	0.668 (0.446)	0.680		
3. Behavioural regulation	0.881	0.714	0.104	0.323 (0.104)	0.276 (0.076)	0.845	
4. Performance failure	0.906	0.658	0.284	0.533 (0.284)	0.532 (0.283)	0.245 (0.06)	0.811

Note. Square root of the AVE in diagonal matrix. CR = composite reliability; AVE = average variance extracted (AVE); MSV = maximum shared variance.

**Table 5:** Model fit indices.

*χ^2^*	*p*	*df*	*χ^2^/df*	GFI	AGFI	CFI	RMSEA
245.9	< 0.000	68	3.616	0.854	0.775	0.901	0.106

*χ^2^* = chi-square; df = degrees of freedom; *χ^2^/df* = chi-square/degrees of freedom; GFI = goodness of fit index; AGFI = adjusted goodness of fit index; CFI = comparative fit index; RMSEA = root mean square error of approximation.

**Table 6:** Total effects between constructs.

Relationship	Sport Anxiety	Burnout	Performance Failure
Behavioural Regulation	0.278 *** *0.278*	0.319 *** *0.134* + (0.278 × 0.666)	0.246 *** *0.093* + (0.278 × 0.551)
Sport Anxiety	------	0.666 *** *0.666*	0.551 *** 0.551

Note. *** *p* < 0.01. Direct effects in italics. Indirect effects in round brackets.

**Table 7:** Mediation role of Sport Anxiety.

Relationship	Direct Effect with Mediator	Indirect Effect
Sport anxiety mediates between behavioural regulation and burnout	0.134	0.185 *** Total
Sport anxiety mediates between behavioural regulation and Performance failure	0.093	0.153 *** Total

Note. *** *p* < 0.01.

## Data Availability

The data presented in this study are available on request from the corresponding author.
